# Effect of diode low level laser and red light emitting diode irradiation on cell proliferation and osteogenic/odontogenic differentiation of stem cells from the apical papilla

**DOI:** 10.1186/s12903-022-02574-8

**Published:** 2022-11-25

**Authors:** Afsaneh Rahmati, Roshanak Abbasi, Rezvan Najafi, Loghman Rezaei-soufi, Hamed Karkehabadi

**Affiliations:** 1 Endodontic Department, School of Dentistry, Hamadan University of Medical Science, Hamadan, Iran; 2 Department of Medical Molecular & Genetics, Faculty of Medicine, Hamadan University of Medical Sciences, Hamadan, Iran; 3 Dental Research Center, Department of Operative Dentistry, School of dentistry, Hamadan University of Medical Science, Hamadan, Iran

**Keywords:** Mesenchymal stem cells, Diode low level laser, Proliferation, Differentiation, LED

## Abstract

**Background:**

This experimental study aimed to assess the effect of irradiation of red light-emitting diode (LED) and Diode low-level laser (LLL) on osteogenic/odontogenic differentiation of stem cells from the apical papilla (SCAPs).

**Materials and methods:**

SCAPs were isolated from the human tooth root. The experimental groups were subjected to 4 J/cm^2^ diode low level laser and red LED irradiation in osteogenic medium. The control group did not receive any irradiation. Cell viability/proliferation of SCAPs was assessed by the methyl thiazolyl tetrazolium (MTT) assay on days 1 and 2 (*n* = 9). Osteogenic differentiation was evaluated by alizarin red staining (ARS) (*n* = 3), and expression of osteogenic genes by real-time polymerase chain reaction (RT-PCR) (*n* = 12) on days 1 and 2. SPSS version 18 was used for data evaluation. The Kruskal-Wallis and Mann-Whitney tests were used to compare the groups at each time point.

**Results:**

The MTT assay showed no significant difference in cell viability/proliferation of SCAPs in the low level laser, red LED, and control groups at 24 or 48 h (*P* < 0.001). The ARS assessment showed that low level laser and red LED irradiation enhanced osteogenic differentiation of SCAPs. low level laser and red LED irradiation both induced over-expression of osteogenic/dentinogenic genes including alkaline phosphatase (ALP), dentin sialophosphoprotein (DSPP), dentin matrix protein 1 (DMP-1), and bone sialoprotein (BSP) in SCAPs. Up-regulation of genes was significantly greater in low level laser irradiation group than red LED group (*P* < 0.001).

**Conclusion:**

Diode low level laser irradiation with 4 J/cm^2^ energy density and red LED irradiation enhanced osteogenic differentiation of SCAPs without adversely affecting cell viability.

## Background

Stem cells from the apical papilla (SCAPs) are mature odontogenic cells that are capable of self-renewal, proliferation, and multipotent differentiation [1, 2]. These cells have significant potential for clinical therapeutic applications and tissue engineering. They have higher proliferation and differentiation capacity than other odontogenic stem cells in vitro [3, 4]. Low level laser (LLL) and light emitting diode (LED) as non-ionizing forms of light are used for photobiomodulation, which has considerable benefits for pain relief, vasodilation, wound healing, and cell proliferation [5, 6]. LLLs have low energy density, and are not associated with heat generation, sound, or vibration [7]. Diode, CO2, and Indium Gallium Aluminium Phosphide (InGaAlP) lasers are among the laser types used for induction of photobiomodulation [8]. Although the exact mechanism of effect of LLL on tissue and cellular biomodulation has yet to be clearly understood, it is known that aside from the cell type and light wavelength, the biological mechanism of LLL depends on activation of enzymes such as cytochrome C oxidase, and proteins such as porphyrins and flavoproteins, photoreceptors, and transcription factors [9, 10]. LED radiation, as a narrow-spectrum safe and effective light source, can effectively stimulate the proliferation and differentiation of cells in vitro, and can serve as a practical tool for induction of tissue regeneration [5]. Only a few in vitro studies are available on the biological effects of LLL or LED on SCAPs [11]. Thus, this study tested the null hypothesis that LLL and red LED irradiation would enhance proliferation and osteogenic/odontogenic differentiation of SCAPs.

## Methods

### Cell culture

The experimental study was performed according to the International Society for Stem Cells Research (ISSCR) Guidelines for Stem Cell Research and Clinical Translation, and approved by the ethics committee of Hamadan University of Medical Sciences (IR.UMSHA.REC.1396.872). SCAPs were isolated from the right upper and lower fully impacted third permanent molars of one healthy donor 18 years of age with an indication for extraction due to orthodontic reasons. Over two-thirds of the right upper and lower third permanent molars root had formed in the donor. The apical papilla tissue was used after obtaining written informed consent from the patient.

Using a dental tweezer, Apical papilla was separated from the apical portion of incompletely developed teeth and the SCAPs were isolated by enzyme digestion and then cultured according to previously reported protocols [4, 12]. A sterile PBS (Phosphate-buffered saline) solution (Gibco BRL, Grand Island, NY, USA) was used to rinse and then store the teeth immediately following extraction. The radicular pulp tissue dissolved in a solution of 3 mg/mL type I collagenase (Worthlington Biomedical, Lakewood, NJ, USA) to collect the stem cells, after that, transferred to Dulbecco’s modified Eagle’s medium (Gibco, GrandIsland, NY, USA) at 37 °C for 1 h. Incubation of the cells was performed at 37 °C, 5% CO2, 85% humidity, and the medium was supplemented with 15% fresh bovine serum, and 1% penicillin and streptomycin. Based on the in vitro study design and the sample size of prior studies [3, 13], three repetitions were conducted for each of the experimental and control groups at each time point.

### Assuring the stemness of cells

Once the cells reached 80% confluence, the culture medium was removed from the flask and the cells were rinsed twice with PBS.

Adding the medium culture to the flask was done after using trypsin/EDTA to detach the cells. The culture medium and the cells were subsequently transferred into a 15-mL Falcon tube and centrifuging at 1200 g for 6 min was done. The cell sediment was rinsed twice with PBS, and flow-cytometry were used to assess the presence of specific stem cell surface markers (CD105 and CD90), and hematopoietic cell surface markers (CD45 and CD34).The cells didn’t exhibit the hematopoietic cell surface markers and were positive for the mesenchymal cell surface markers.

### Study group

The cells were evaluated in three groups:

- low level laser irradiation (LLLI), red LED irradiation, and non-irradiating cells (control).

### Irradiation of diode low level laser (epic10, BIOLASE, Inc., Irvine, CA, USA)

Diode low level laser (epic10, BIOLASE, Inc., Irvine, CA, USA) at 940 nm wavelength and 50 mW power was used in this study. The cells were irradiated in 24-well plates in standard mode in the dark. Laser was irradiated directly, perpendicular to the cell culture. Time was adjusted to reach 4 J/cm^2^ fluence. First irradiation was performed at 24 h after the primary cell culture and was repeated at 48 h. Cell proliferation was evaluated in the irradiated and control groups at 24 and 48 h.

### Irradiation of red LED

A red LED irradiation (Fotosan 630, Korea, MDD, CMS Dental Denmark) operating at 640 nm wavelength (1 W output) was used in this study. The distance between the light source and cell layer was 1 cm, the spot size was 3.5 cm, and the irradiation was performed for 30 s. The power density at the cell surface was 100 mW/cm^3^.

According to previous studies [14], the respective formula (energy density = power density x irradiation time), and the expected energy level. The first irradiation was performed at 24 h after the primary cell culture, and repeated at 48 h. All irradiations were performed by the same operator.

### Non-irradiating cells (control group)

The control group includes non-irradiated control cells exposed to room light for the same period and maintained under the same conditions as irradiated cells. Cell viability/proliferation in the control and irradiated groups was evaluated after 24 and 48 h.

### Assessment of the viability and proliferation of SCAPs

Cell viability/proliferation was evaluated using the methyl thiazolyl tetrazolium (MTT) assay. For this purpose, 5 × 10^3^ cells were plated in 96-well plates. After 24 and 48 h, 10 λ of the MTT solution was added to all wells, and the plates were incubated at 37 °C. After 3 h, the contents of the wells were removed, and 100 λ dimethyl sulfoxide was added to the sediment. Optical density was read by an ELISA plate reader (Bio-Rad 680, USA) at 570 nm wavelength. Three samples of each study groups were prepared and analyzed (*n* = 9).

### Assessment of osteogenic/odontogenic differentiation by real-time polymerase chain reaction (PCR)

Total RNA was extracted from SCAPs in all experimental and control groups, and the expression of several odontogenic/osteogenic genes was evaluated using real-time PCR. Total RNA was extracted using TRIzol (Invitrogen, CA, USA) according to the manufacturer’s instructions. The cDNA was then synthesized using the cDNA synthesis kit (Superscript II first-strand cDNA synthesis kit, Invitrogen, CA, USA) according to the manufacturer’s instructions. Real-time PCR was performed using 7500 Fast Real-Time PCR (Applied Biosystems، Carlsbad, CA, USA). Table 1 shows the sequence of primers used for the genes. Real-time PCR was applied to assess the expression of Alkaline phosphatase (ALP), dentin sialophosphoprotein (DSPP), dentin matrix protein 1 (DMP-1), and bone sialoprotein (BSP) in each cell group. The experiment was repeated three times for each gene (*n* = 12).

**Table 1:** Primers sequences used for quantitative RT-PCR

Genes		Primer sequences
DSPP	Forward	GCTGGCCTGGATAATTCCGA
	Reverse	CTCCTGGCCCTTGCTGTTAT
DMP1	Forward	5 -CAACTGGCTTTTCTGTGGCAA-3
	Reverse	5 -TGGGTGCGCTGATGTTTGCT-3
BSP	Forward	TTTCCAGTTCAGGGCAGTAGTGACT
	Reverse	AGGCGTGGCGTCCTCTCCATAG
ALP	Forward	GACCTCCTCGGAAGACACTC
	Reverse	TGAAGGGCTTCTTGTCTGTG

*DSPP* dentin sialophosphoprotein, *DMP1* dentin matrix protein 1, *BSP* bone sialoprotein, and *ALP* alkaline phosphatase

### Alizarin red staining (ARS)

The odontogenic/osteogenic differentiation of SCAPs was evaluated following photobiomodulation interventions in a 24-well plate containing odontogenic medium. The odontogenic medium [15, 16] was prepared by adding 10 mM beta glycerophosphate (Sigma-Aldrich, St. Louis, MO, USA), 100 nm dexamethasone (Sigma-Aldrich, St. Louis, MO, USA), and 50 mg/mL ascorbic acid into the growth medium, which was refreshed every 72 h. The cells were fixed with 4% paraformaldehyde and rinsed with phosphate buffered saline after 21 days, and formation of mineralized calcium nodules was evaluated by using 1% ARS solution (Sigma-Aldrich, St. Louis, MO, USA) and incubation at 37 °C for 30 min [17]. The experiment was repeated three times (*n* = 3) .The results were spectrophotometrically (SpectraMax 2 M) analyzed at 562 nm wavelength.

### Statistical analysis

Data were analyzed using SPSS version 18. The Kruskal-Wallis and Mann-Whitney tests were used to compare the groups at each time point.

## Results

### Results of MTT assay

**Fig. 1:**
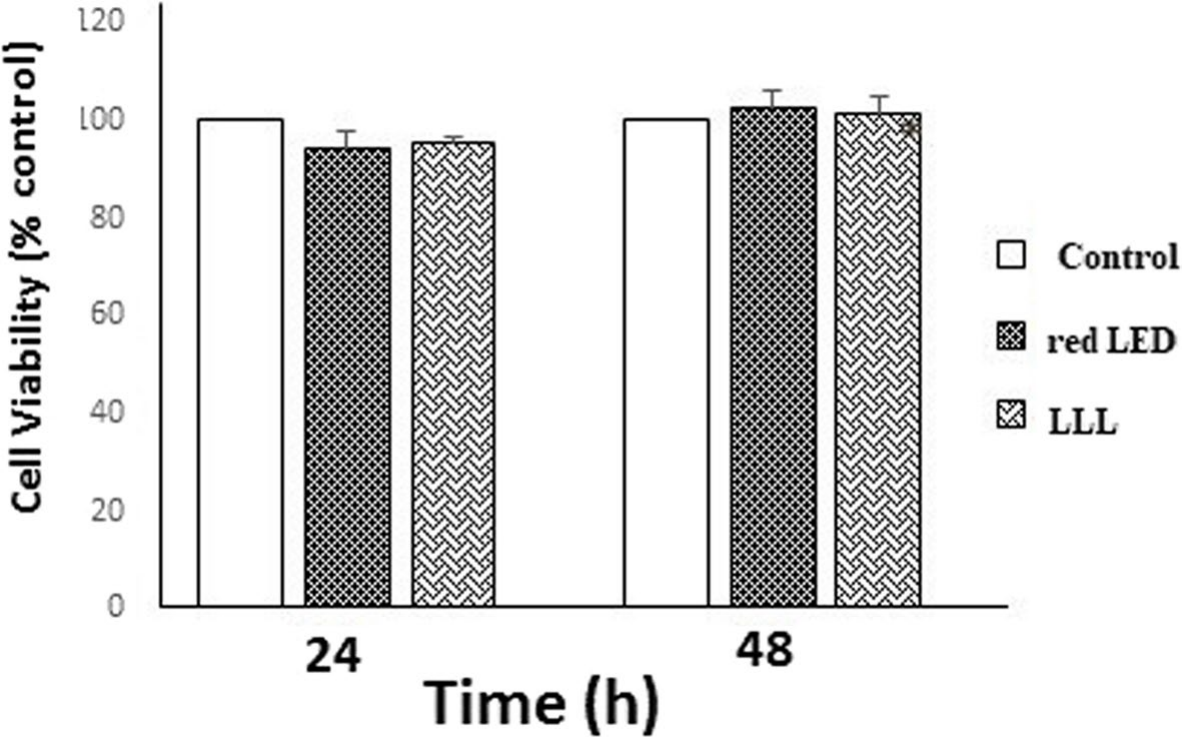
Cell viability of SCAPs (MTT assay) subjected to LLL, red LED, and control groups at 24 and 48 h. At 24 and 48 h, there was no significant difference in proliferation/viability of SCAPs between the LLL, red LED, and control groups in MTT assay

The MTT assay found no significant difference in proliferation/viability of SCAPs among the LLL, red LED, and control groups at 24 or 48 h (*P* > 0.05, Fig. 1).

### Results of real-time PCR to assess the osteogenic/odontogenic differentiation potential of SCAPs

**Fig. 2:**
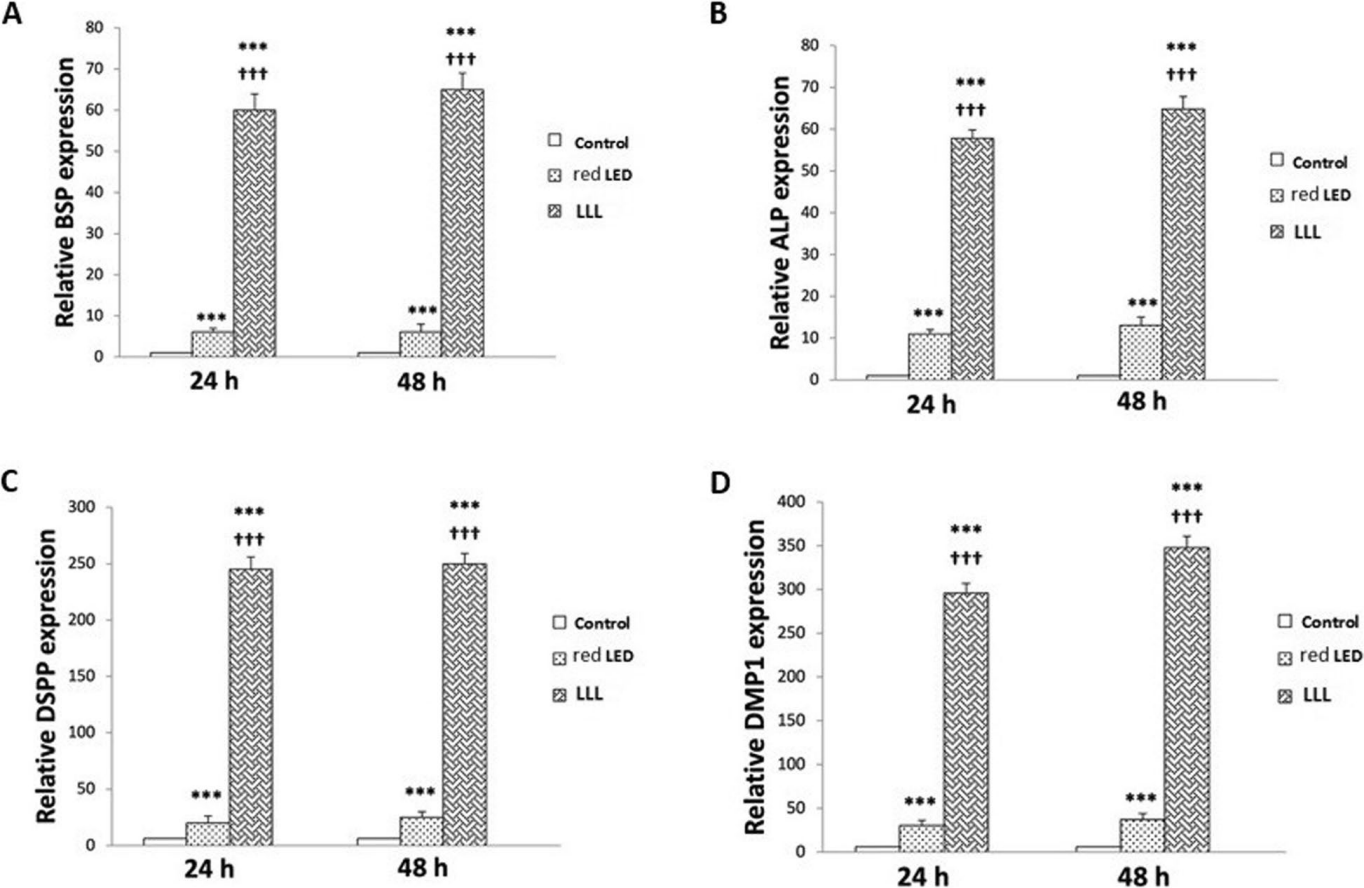
The effects of red LED and low level laser on osteogenic and odontogenic differentiation of SCAPs, relative mRNA expression of genes: **A**: BSP, **B**: ALP, **C**: DSPP, **D**: DMP1, after 24 and 48 h, the expression of BSP (**A**), ALP (**B**), DSPP (**C**), and DMP1 (**D**) genes in the LLL and red LED groups was significantly higher than that in the control group, and the expression of BSP (**A**), ALP (**B**), DSPP (**C**), and DMP1 (**D**) genes in the low level laser group was significantly higher than that in the red LED group (*P* < 0.001)

To assess the effect of LLL and red LED irradiation on osteogenic/odontogenic differentiation of SCAPs, the expression level of mRNA of several genes related to osteogenic/odontogenic differentiation of SCAPs namely BSP, DSPP, ALP, and DMP1 was evaluated by real-time PCR. The expression of each gene in the experimental groups was compared with the corresponding value in the control group. As shown in Fig. 2, after 24 and 48 h, the expression of BSP (Fig. 2A), ALP (Fig. 2B), DSPP (Fig. 2C), and DMP1 (Fig. 2D) genes in the LLL and red LED groups was significantly higher than that in the control group. Moreover, the expression of BSP (Fig. 2A), ALP (Fig. 2B), DSPP (Fig. 2C), and DMP1 (Fig. 2D) genes in the low level laser group was significantly higher than that in the red LED group (*P* < 0.001).

### Alizarin red staining (ARS)

**Fig. 3:**
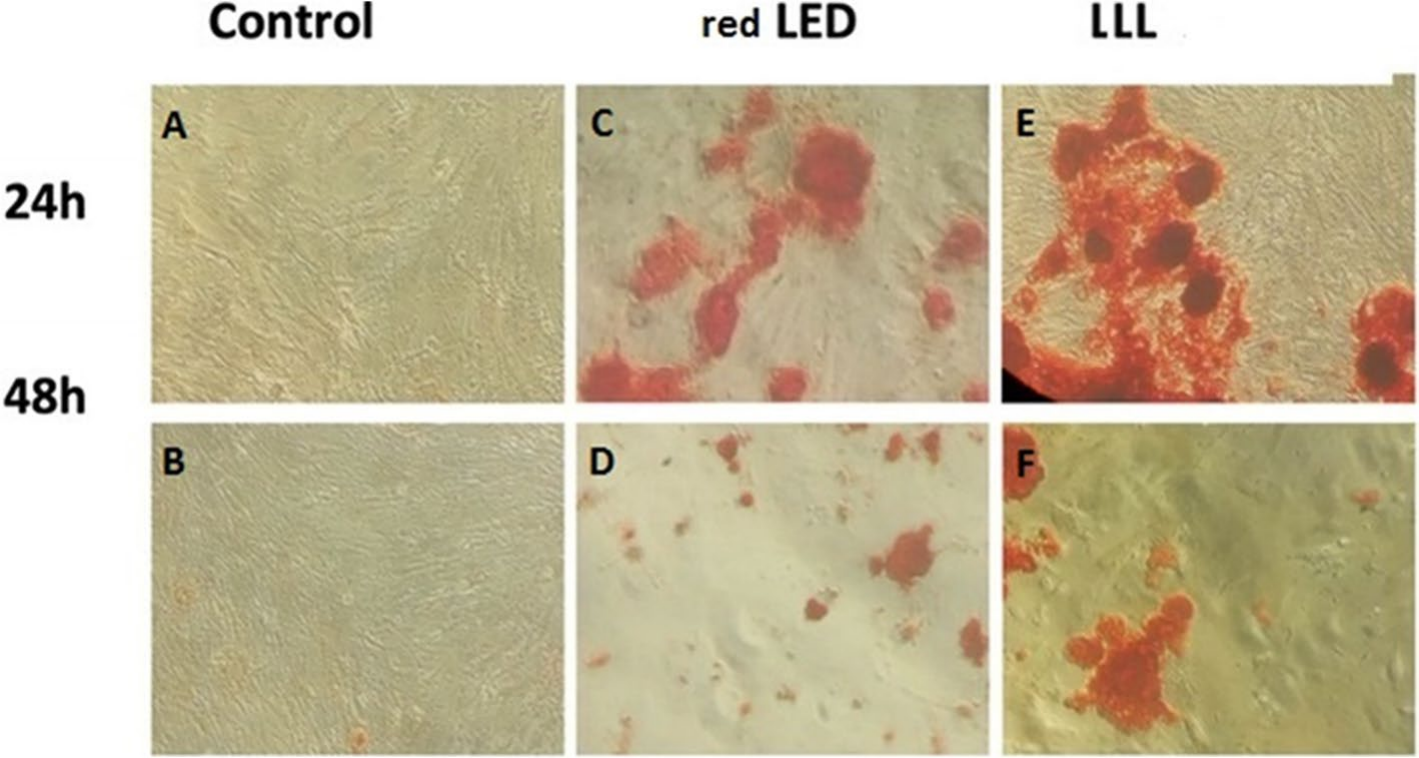
Qualitative evaluation of osteogenic differentiation of SCAPs, The SCAPs were stained with 1% alizarin red to detect mineralized nodules, Density of ARS in the control (**A**, **B**), red LED (**C**, **D**), and LLL (**E**, **F**) groups after 24 and 48 h. A significant increase in osteogenic mineralization was observed in the LLL and red LED groups. Maximum increase was noted in the laser group. There was a smaller increase at 48 h than at 24 h

Osteogenic differentiation of SCAPs following low level laser or red LED irradiation was also evaluated qualitatively by ARS, which is the most recent osteogenic index showing matrix mineralization. As shown in Fig. 3, the ARS density in both red LED and LLL groups was higher than that in the control group at 24 and 48 h. In other words, both red LED and low level laser irradiation increased osteogenic mineralization. Moreover, maximum increase was noted in the low level laser group. Additionally, there was a smaller increase at 48 h than at 24 h.

## Discussion

It has been well confirmed that SCAPs have proliferation and differentiation potential comparable to that of neural crest cells [18, 19]. SCAPs originate from the primary odontoblasts, and are imperative for root dentin formation and root development [20, 21]. Formation of pulp-dentin complex, differentiation to osteoblast-like cells, and strong resistance against infection and inflammation are among the unique properties of SCAPs. Such properties highlight their significant role in root development, and osteogenic and odontogenic regeneration [5].

In vitro evidence shows that SCAPs are superior to other odontogenic stem cells in terms of proliferation and differentiation [22]. In the clinical setting, photobiomodulation with appropriate laser parameters may serve as an adjunct, and improve clinical results [23]. Several studies have shown significant effects of LLLs on proliferation and differentiation of different cell types such as mesenchymal cells, cardiac stem cells, and other mature cells such as fibroblasts and endothelial cells [24]. A study on the effects of LLL on dental pulp stem cells, SCAPs, periodontal ligament stem cells, and stem cells from human exfoliated deciduous teeth discussed that limited evidence exists supporting the hypothesis that LLL irradiation enhances the proliferation of mesenchymal stem cells [25]. Different laser types, mainly He-Ne diode laser and InGaAlP laser with 660, 810, and 980 nm wavelengths and energy density of 0.1 to 230 J/cm^2^ have been used in the literature. There have been several papers stating that the practical treatment window for photobiomodulation therapy for osteogenic/odontogenic differentiation of stem cells is between 600 and 900 nm. On the basis of this spectrum, we investigated the efficacy of the low level laser with the wavelength of 940 nm, which is not too far off. [5] [26–28]. The energy density selection criteria was based on the average of the most widely used energy density as indicated in systematic reviews [23, 26, 29, 30]. Additionally, we hypothesized that the energy density of 4 J/cm2 would be more effective based on these articles [31–33]. As a final note, The results of our study were consistent with those of the articles cited above.

The present study is the first in vitro study that comprehensively assessed the effect of diode low level laser and red LED on biological endpoints related to proliferation and osteogenic/odontogenic differentiation of SCAPs. LED irradiation has antibacterial effects and also increases the expression of markers of differentiation of keratinocytes [5]. The energy density of red LED may range from 0.5 to 10 J/cm^2^ and has significantly different effects on cells [7]. One study showed that energy density of 4 to 5.5 J/cm^2^ was more effective in induction of proliferation of stem cells [34]. Also, it has been reported that red LED at 600–700 nm wavelength can affect the proliferation and differentiation of mesenchymal stem cells in amniotic fluid [35]. Considering the significance of SCAPs in osteogenic and odontogenic differentiation, this study assessed the effect of red LED and laser on proliferation and osteogenic/odontogenic differentiation of SCAPs. In the present study, diode low level laser at 940 nm wavelength with maximum power of 50 mW was used in continuous-wave mode with 4 J/cm^2^ fluence, which had no significant effect on cell viability/proliferation at 24 and 48 h as shown by the MTT assay. Gholami et al. [36], in there study, used a 940 nm InGaAs Semi-conductor diode laser (EpicX, Biolase, USA) in three sessions with 48 h intervals to evaluate the effect of photobiomodulation on periodontal ligament stem cells, similarly to our results, the MTT assays showed no significant difference between the laser-irradiated group and controls at any of the time points. Pereira et al. [17] found no significant difference in proliferation and differentiation of human dental pulp stem cells between the laser and control groups. Eduardo et al. [37] used 660 nm laser with 3 J/cm^2^ fluence and 20 mW power and showed that its effect on cell proliferation (assessed by the MTT assay) at 72 h was significantly higher than the effect of laser with 40 mW power and the control group. Irradiation at 630–670 nm wavelength and 0.5-3 J/cm^2^ fluence also showed a positive effect on proliferation and differentiation of bone marrow mesenchymal stem cells [38]. Wu et al. [39]showed that 660 nm LLL irradiation with three different energy densities of 1, 2 and 4 J/cm^2^ significantly increased the proliferation of mesenchymal stem cells irrespective of energy density. These results confirmed the more favorable effect of laser with lower power on cells which enabled them to irradiate the cells for a longer period than we did. Although LLL in visible wavelength spectrum is often irradiated on stem cells, infrared radiation with 805–810 nm wavelength and different energy densities has also been suggested for this purpose [40, 41]. Other studies on the effects of LLL irradiation on proliferation of human osteoblasts [42] ,human mesenchymal stem cells [26], fibroblasts [43], bone marrow stem cells [44] and epithelial cells [45] reported similar findings.

In the present study, the MTT assay was used for evaluation of cell viability/proliferation due to its optimal accuracy and availability. The effect of red LED irradiation on proliferation and differentiation of SCAPs was evaluated at 24 and 48 h. The results of the MTT assay revealed no significant effect of red LED irradiation on proliferation and viability of SCAPs. Our results was different from the findings of a previous study that used LED with 0–4 J/cm^2^ energy density, and showed an inhibitory effect of the low energy blue LED on the proliferation of gingival mesenchymal stem cells [46]. Although, Yang et al. [5] reported that the proliferation rate was lower in laser groups than the control group. They demonstrated that LED irradiation inhibited the proliferation of SCAPs in osteogenic medium. Evidence shows that the best results in this respect may be obtained by the use of light in visible spectrum (600–700 nm) [47]. In contrast, irradiation of light in infrared spectrum (810–830 nm) is correlated with inhibition of gene transcription [27, 48]. Overall, Different power outputs, fiber distances from the monolayer, irradiation times, continuous or pulsed mode of irradiation, and cell type could explain this inconsistency.

Odontogenic regeneration starts with odontoblastic differentiation. The expression of osteoblastic genes is induced after the differentiation of mesenchymal stem cells [49]. BSP, DMP-1, DSPP, and ALP genes are osteoblastic markers that are closely correlated with osteoblastic differentiation [5, 50]. DSPP is a marker of odontoblastic differentiation, and plays an important role in coding of DSPP and dentin sialoprotein [3]. DMP-1 is an extracellular matrix glycoprotein that plays an active role in odontoblastic differentiation, and is an imperative component in dentin mineralization [3].

The results of real-time PCR on SCAPs in the present study revealed that the expression of BSP, DSPP, DMP-1 and ALP that are correlated with osteogenic/odontogenic differentiation of SCAPs significantly increased in groups subjected to LLL or red LED irradiation. Moreover, the expression of these genes in the LLL group was significantly greater than that in the red LED group. These results are in line with those of a review study on the effect of LLL on proliferation of dental pulp stem cells. In the above mentioned study, InGaAlP laser at 660 nm wavelength and 3 J/cm^2^ energy density increased cell proliferation compared with the control group [7]. The high osteogenic potential of SCAPs in comparison with DPSCs and BMSCs has been shown in previous studies [3]. Turrioni et al. reported a significant increase in ALP activity and collagen synthesis as well as the expression of DSPP (2 J/cm^2^), COL-1, DMP-1 (4 J/cm^2^), and ALP (4 J/cm^2^) in stem cells from human exfoliated deciduous teeth and dental pulp [51].

According to the result of this present study alizarin red staining showed higher deposition of calcium nodules with significant differences in both LLLI and red LED groups in comparison with the control group (*P* < 0.05). The LLLI and red LED enhanced the osteogenesis over SCAPs in vitro because laser irradiation could modulate SCAPs osteogenic differentiation significantly. Different studies have also proven low level laser therapy’s (LLLT) capability of improving osteogenic differentiation of different mesenchymal stem cells [5, 33]. Although the detrimental effect of blue LED irradiation on some types of stem cells has been reported [52]. Based on assessment of Alizarin Red staining, we observed a smaller increase at 48 h than 24 h. The reason for this may be the saturation of osteogenic induction due to the limited cell growth areas, cell maturation, and the switch from bone formation processes by osteoblasts to bone maintenance processes by osteocytes [39, 53].

### Limitations and sights for future studies:

It is imperative to identify the optimal characteristics of the LLLI setting (energy density, energy output, distance between the cells and the laser spot/probe) in order to increase the dental stem cells proliferation in clinical practice and determine the feasibility of its use. Based on data available in systematic reviews [23, 26], most studies used 6–48 h intervals to irradiate stem cells, however, due to financial limitations, we used a short time interval (24, 48 h) in this study. In future studies, longer time intervals (72, 96, 168 h) would be evaluated.Since the apical zone of the tooth contains a small number of stem cells, cell expansion is required to obtain adequate numbers of cells for clinical use. [54]. The difficulty in producing differentiated cells from SCAPs results in limitations in providing the cells for injured tissue within a short period of time [55]. Considering cell migration, homing, and apoptosis are other factors that should be considered when assessing the effects of treating SCAPs with LLL. However, the exact mechanisms by which LED and LLL irradiation impacts the proliferation of SCAPs and their differentiation into osteogenic and odontogenic cells remain unclear and more research is required.

## Conclusion

Based on the results of this study, diode low level laser irradiation with 4 J/cm^2^ energy density and red LED enhanced osteogenic differentiation of SCAPs without adversely affecting cell viability. This study also suggests that LED irradiation and LLLI can be added to stem cell-based treatments such as regenerative endodontic treatments.

## Data Availability

The complete documentation of participants enrolled in this study belongs to the corresponding author, Hamed Karkehabadi, and are available only upon reasonable request.

## Abbreviations

SCAPs: Stem cells from the apical papilla
MTT: Methyl thiazolyl tetrazolium
InGaAlP: Indium Gallium Aluminium Phosphide
ISSCR: International Society for Stem Cells Research
qrt-PCR: Quantitative reverse-transcription polymerase chain reaction
ARS: Alizarin red staining
LLL: Low level laser
LLLI: Low level laser irradiation
LLLT: Low level laser therapy
ALP: Alkaline phosphatase
DSPP: Dentin sialophosphoprotein
DMP1: Dentin matrix protein 1
BSP: Bone sialoprotein
DPSCs: Dental pulp stem cells
hPDL: Human periodontal ligament
hPDLSCs: Human periodontal ligament stem cells
LED: Light emitting diode
PBS: Phosphate-buffered saline
SPSS: Statistical Package of the Social Sciences
